# Distance Monitoring of Advanced Cancer Patients with Impaired Cardiac and Respiratory Function Assisted at Home: A Study Protocol in Italy

**DOI:** 10.3390/jcm12051922

**Published:** 2023-02-28

**Authors:** Rita Ostan, Silvia Varani, Andrea Giannelli, Italo Malavasi, Francesco Pannuti, Raffaella Pannuti, Guido Biasco, Anna Vittoria Mattioli, on behalf of the ANT Collaborative Group

**Affiliations:** 1 Training and Research Department, National Tumor Assistance (ANT) Foundation, Via Jacopo di Paolo, 36, 40128 Bologna, Italy; 2 Nethical S.r.l., Via Paolo Fabbri, 1/4, 40138 Bologna, Italy; 3 Department of Experimental, Diagnostic and Specialty Medicine, University of Bologna, Via Giuseppe Massarenti, 9, 40138 Bologna, Italy; 4 Department of Medical and Surgical Sciences for Children and Adults, University of Modena and Reggio Emilia, Via del Pozzo, 71, 41124 Modena, Italy

**Keywords:** advanced cancer patients, telemedicine, telemonitoring, cardio-oncology, home care

## Abstract

During the pandemic, telemedicine and telehealth interventions have been leading in maintaining the continuity of care independently of patients’ physical location. However, the evidence available about the effectiveness of the telehealth approach for advanced cancer patients with chronic disease is limited. This interventional randomized pilot study aims to evaluate the acceptability of a daily telemonitoring of five vital parameters (heart rate, respiratory rate, blood oxygenation, blood pressure, and body temperature) using a medical device in advanced cancer patients with relevant cardiovascular and respiratory comorbidities assisted at home. The purpose of the current paper is to describe the design of the telemonitoring intervention in a home palliative and supportive care setting with the objective of optimizing the management of patients, improving both their quality of life and psychological status and the caregiver’s perceived care burden. This study may improve scientific knowledge regarding the impact of telemonitoring. Moreover, this intervention could foster continuous healthcare delivery and closer communication among the physician, patient and family, enabling the physician to have an updated overview of the clinical trajectory of the disease. Finally, the study may help family caregivers to maintain their habits and professional position and to limit financial consequences.

## 1. Introduction

The overloading of healthcare facilities and the necessity to prevent the risk of infection during the COVID-19 emergency have highlighted the need to implement significant changes in the organization and delivery of healthcare services, mainly when these are aimed at the most fragile segments of the population [1,2,3]. Within this context, telemedicine and telehealth interventions have acquired a leading role in maintaining the continuity of care independently from the physical location of cancer patients. However, the evidence available about the effectiveness of the telehealth approach for advanced cancer patients with chronic disease is limited [4,5].

Once the emergency was addressed, the attention to telemedicine interventions has remained high, although preliminary evidence is mixed and greatly variable depending on the type of disease [6].

As concerns patients with advanced cancer, a recent systematic review studied the degree of efficacy regarding the use of diverse web and technological devices, finding positive effects on quality of life (QOL) and psychosocial well-being [7]. However, the level of user satisfaction and engagement with these interventions remains unclear for advanced cancer patients. Recent research suggested that patient engagement can be considered a key factor in the effectiveness evaluation and a primary criterion in feasibility studies of telehealth approaches [8]. Therefore, there is a need to further investigate how advanced cancer patients engage with the wide range of existing telehealth devices to offer patient-centered care with a telemonitoring intervention personalized based on patients’ needs and clinical condition

Furthermore, scientific evidence is progressively underlining the potential role of telemedicine in home care protocols to assist an increasing number of advanced cancer patients with an efficient program of early palliative care [9,10]. The introduction of telemonitoring systems aims to facilitate more and more effective patient care, reducing both emergency room and hospital admissions and improving symptoms of distress, anxiety, and depression in patients and caregivers [11,12,13]. Self-monitoring also increases patients’ awareness by making them feel part of their care pathway and active contributors to their well-being [14,15,16].

Telemonitoring mainly uses medical devices to record parameters relating to the patient’s state of health or treatment progress. Once registered, the data are shared on a cloud platform and then viewed by the physician, who can intervene in real time in the event of clinically significant anomalies reported by the system.

Advanced cancer patients frequently have multiple comorbidities (i.e., respiratory distress, cardiovascular disease) due to disease progression and/or the side effects of therapies [17,18]. The presence of comorbidity makes this population even more fragile and needs fast and successful management of symptoms as well as integration and sharing among the specialists treating the patient, including oncologists and cardiologists [19]. Indeed, telemedicine and telemonitoring systems may support collaboration among health professionals, communication among health professionals and patients, as well as patient/family self-care in a patient empowerment mode [20].

The present study is embedded in the framework of the home palliative and supportive care program provided by the National Tumor Assistance (ANT) Foundation [21,22]. The mission of the ANT Foundation is to offer the possibility of spending the last period of life in one’s living environment, envisaging not only the physical effects of the disease but also the patient’s well-being and perceptions, with the main objective of preserving the quality of life of the patients and their families. To this aim, a multidisciplinary équipe of physicians, nurses, psychologists, and volunteers assists patients and their families at home during the different stages of the cancer disease, ensuring respect for their privacy, dignity, and autonomy.

Over the past few years, the ANT Foundation has introduced new technologies into its care space. Specifically, ANT introduced the use of virtual reality into clinical practice as a complementary therapy to improve the quality of life and the psychophysical symptoms experienced by the patients. The studies conducted by the ANT Foundation showed that the virtual reality intervention in oncological patients cared for at home had a positive impact on the short-term symptoms experienced by patients, inducing a decrease in pain, depression, anxiety, and shortness of breath and an improvement in well-being [23,24].

In addition, to meet the challenges imposed by the COVID-19 pandemic, ANT implemented audio and video call visits in order to continue to support patients and their families even at a distance. Specifically, Franchini et al. (2021) suggested that online visits should progressively be integrated into medical practice, although they should not replace visits in person [25].

This interventional study aims to evaluate the acceptability of the daily telemonitoring of five vital parameters (heart rate, respiratory rate, blood oxygenation, blood pressure, and body temperature) using a medical device in advanced cancer patients with relevant cardiovascular and respiratory comorbidities assisted at home.

The purpose of the current paper is to describe the design of this telemonitoring intervention in a home palliative and supportive care setting with the objective of optimizing the management of patients, taking into account not only their quality of life and psychological status but also the caregiver’s perceived care burden and satisfaction with the received care.

## 2. Materials and Methods

### 2.1. Study Design

**Figure 1:**
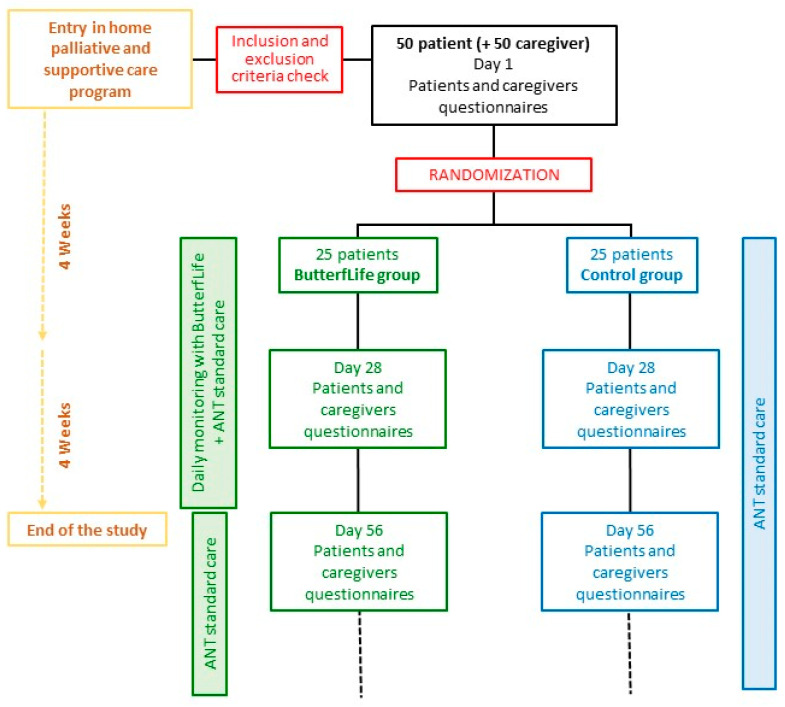
Flow diagram of the study. ANT: National Tumor Assistance Foundation.

The proposed investigation is a non-profit, single-center, interventional randomized pilot study with a medical device without drugs (Figure 1). The trial was registered at clinicaltrials.gov (accessed on 20 December 2022) (NCT05650814).

### 2.2. Setting

The home care model managed by the ANT Foundation employs a hospital-at-home approach in which a multidisciplinary team of physicians, nurses and psychologists, all trained in palliative and supportive care, works around-the-clock 24 h/7 days a week to assist cancer patients. The service is free for the patients, and it is offered in agreement with the National Health Service [21,22].

### 2.3. Study Population

Participants are advanced cancer patients assisted within the home care program of the ANT Foundation in the metropolitan area of Bologna (Italy). Inclusion and exclusion criteria can be found in Table 1.

### 2.4. Recruitment and Randomization

The investigator will be the ANT physician in charge with the home care of the patient. During the first visit, after verifying the inclusion and exclusion criteria, the investigator (ANT physician) will explain the study to the patient and caregiver and propose their participation. If they consent, after collecting informed consent for participation and personal data management, the physician will contact the Research Department of the ANT Foundation to include the dyad (patient and caregiver) in the study.

Participants will be randomly assigned by the Research Department staff to the ButterfLife group (25 patients) or the Control group (25 patients) by randomization using an Excel-generated random number set. Investigators that will run the statistical analysis will be blinded to the randomization procedure.

### 2.5. Procedures and Data Collection

The study will last eight weeks for each patient. On the first day (day 1), the investigator will ask the patient and his/her caregiver to answer the questionnaires.

**Figure 2:**
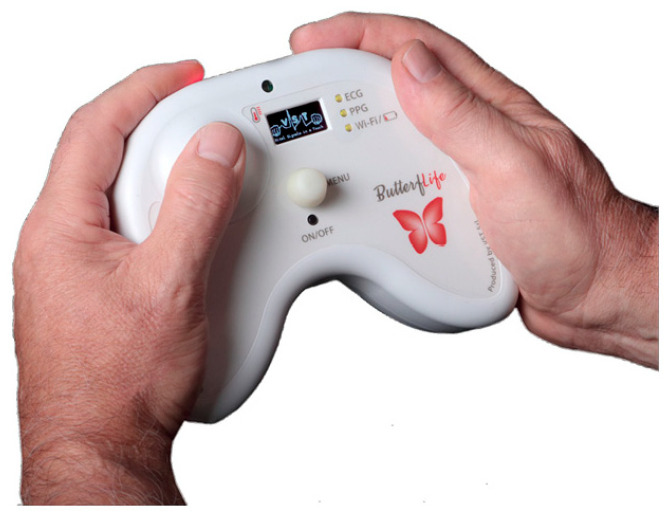
ButterfLife medical device.

For patients in the ButterfLife group, the investigator will deliver the ButterfLife device (Figure 2) at home and explain its use. ButterfLife is a medical device similar to a joypad, designed by the Unimore spinoff VST (Vital Signal in a Touch, VST Srl, Modena, Italy), which, through the use of three different sensors, a photoplethysmogram, electrocardiogram, and an infrared thermometer, simultaneously measures the five primary vital parameters defined by the World Health Organization in 90 s (heart rate, respiratory rate, blood oxygenation, blood pressure, and body temperature). The ButterfLife device is designed to make self-measurements and therefore requires no external intervention for measurements. To record these parameters, the patient has to place both hands on the device, and the data collected are immediately transmitted to a portal whose software (VST-FIVE) calculates the vital signs and allows visualization of the sharing with the doctor via a Wi-Fi network (https://my.vital.st/) (accessed on 22 December 2022). To guarantee a stable internet connection, a 4G LTE Wi-Fi Router (TP-Link, Hong Kong) will be provided at the patient’s home. In this way, it is possible to ensure the early detection of worsening and failure of the health state, enabling the timely activation of appropriate intervention in the home setting. The recorded data remains available for subsequent evaluation, long-term monitoring, and offline analysis. The patient is recommended to take the measurement once a day, preferably in the morning, and to repeat the measurement of parameters during the day if necessary. The investigator will leave a booklet containing simple written instructions with images explaining the use of ButterfLife. The doctor will check the data recorded by the patient every day (Supplementary Document 1) and the trend of the parameters over time by a graphical representation on the electronic medical record (Vitaever, Nethical Srl, Bologna, Italy) (https://www.vitaever.com/) (accessed on 20 December 2022). Daily telemonitoring will be conducted in addition to standard home care.

Patients in the control group will receive standard home care: visits will be planned according to the patient’s needs. In case of an unstable clinical condition, worsening of symptoms, and alteration of vital parameters, the patient can contact the physician or the nurse by phone, who will decide how to intervene.

### 2.6. Measures and Outcomes

The primary outcome of the study is the acceptability and the feasibility of the telemonitoring intervention measured as the percentage of eligible patients proposed for the study who agree to participate and the number of self-measurements/week per patient. Moreover, a questionnaire for evaluating the ButterfLife device (ease of use, difficulties encountered, and usefulness) was constructed based on several questionnaires already found in the literature [26] and will be administered to patients belonging to the ButterfLife group at the end of the telemonitoring period.

The secondary outcomes involving patients and caregivers are listed below.

- The quality of life (QOL) of patients will be assessed by the EuroQoL-5D-3L questionnaire (EQ-5D-3L), Italian version [27,28], a generic instrument consisting of 2 distinct sections: patient’s subjective assessment of five dimensions of quality of life (mobility, self-care, daily activities, pain/discomfort, and anxiety/depression); each item has responses graded from 1 to 3, where level 1 indicates no problems and level 3 indicates extreme limitation. Assessment of the patient’s perceived health status will be made on a visual analogue scale (VAS) graded from 0 (the worst possible health status) to 100 (the best possible health status).
- The psychological status of the patient will be evaluated by the Depression Anxiety Stress Scales-21 (DASS-21), Italian version [29,30], a self-assessment scale to detect depression, anxiety and stress. The scale consists of 21 items, 7 for each emotional state, assessed on a 4-point Likert scale (from 0 = never to 3 = always).
- Care burden perceived by the caregiver will be assessed by the Caregiver Burden Inventory (CBI) [31], a questionnaire consisting of 24 questions on 5 domains (time-dependent care burden, developmental burden, physical burden, social burden, and emotional burden). Completion requires ticking the box from 0 to 4 (0 = not at all, 1 = slightly, 2 = moderately, 3 = quite a lot, 4 = very much) that best describes the current condition or personal impression of the caregiver. The total score ranges from 0 to 100, where 100 indicates the highest perceived caregiver burden.
- Satisfaction with care stated by the caregiver will be assessed through the Family Satisfaction with Advanced Cancer Care-2 questionnaire (FAMCARE-2), Italian version [32,33], a specific validated instrument to measure family members’ satisfaction with the care received from a palliative care team for their relative with advanced cancer. The FAMCARE-2 consists of 17 questions whose answers, on a 5-point ordinal score ranging from 1 (very dissatisfied) to 5 (very satisfied), cover 5 domains (management of symptoms and patient comfort, information provided, support to the family, and psychological assistance to the patient). The total FAMCARE-2 score can range from 17 to 85, where 85 represents the highest satisfaction with the care received.

The total monitoring time will be 8 weeks: at day 1, after 4 weeks (day 28), and 8 weeks (day 56), the administration of questionnaires to the patient and caregiver will be repeated (Table 2).

Moreover, a series of secondary outcomes will be evaluated on physicians, the ANT Foundation, and the National Health System:

- The acceptability and ease of use of the device will be assessed by a questionnaire directed to the physician.
- The number of scheduled, unscheduled, and on-call visits (doctor and nurse), as well as the number of phone calls and emergency phone calls received by the doctor and nurse, will be assessed.
- The number of admissions to the emergency room will be assessed.
- Hospitalization days will be assessed.

### 2.7. Other Assessments

All the clinical and demographic data of patients, as well as the health care services provided by ANT physicians and nurses, will be collected on an electronic record form exploiting Cloud technology created by Vitaever SaaS (Nethical S.r.l., Bologna, Italy) (https://www.vitaever.com/) (accessed on 15 December 2022).

Data related to patients entering the study will be extracted by the Vitaever database in Excel format. The following data will be used in the study:

- Demographic data (sex, age).
- Primary site of disease (recorded according the International Classification of Diseases (9th revision) [34] and then classified as gastrointestinal, respiratory tract, genitourinary, breast, nervous system, haematological, and other), time since diagnosis, and presence of metastases.
- Disease stage (radically operated cancer (adjuvant therapy—supportive therapy); advanced cancer (early palliative care—supportive therapy); locally advanced cancer (adjuvant therapy); advanced cancer (palliative care only).
- KPS.
- Edmonton Symptom Assessment Scale (ESAS) [35].
- Cancer therapy (chemotherapy, hormone therapy, radiotherapy, immunotherapy) and pain therapy.

### 2.8. Training and Monitoring

In order to standardize the process among the physicians participating as investigators, the questionnaires and the study procedures were practiced and discussed during a training session with the Research Department staff prior to the start of the recruitment period. Moreover, the investigators were trained by the VST technicians in the use of the ButterfLife device and in the consultation of the physiological parameter reports.

Periodic meetings between the investigators and the Research Department staff have been scheduled to monitor the progress of the study and discuss the difficulties encountered.

### 2.9. Sample Size

Based on data from patients entering care in 2020, it is planned to propose the study to approximately 60 patients meeting the inclusion and exclusion criteria. Among these, it is supposed that at least 50 patients will agree to enter the study and be randomized into two groups (ButterfLife and Control) of 25 patients each.

The power of the study, calculated using the G*Power software with a post hoc power analysis for a Mann–Whitney test between two groups counting on an effect size = 0.8 and α = 0.05, was (1 − β) = 0.86. Therefore, 50 patients cared for at home by the ANT Foundation in Bologna and its province will be included in the study.

### 2.10. Statistical Analysis

A statistical analysis will be conducted on the data after their anonymization. An investigator is in charge of anonymization and blinding to groups’ patient allocation. The anonymized and blinded data will be passed to the investigators in charge of data analysis. Anonymized data will be stored within the Research Department of the ANT Foundation in an ad hoc safe electronic database. The statistical analysis will be structured as follows:

- Descriptive analysis of the characteristics of the recruited subjects. The following parameters are shown as frequencies: age, sex, KPS, diagnosis, time since diagnosis, presence of metastases, symptoms, current therapies, and stage of the disease.
- Analysis of acceptability and feasibility: percentage of eligible patients proposed for the study who agree to participate, percentage of drop out from the study, number of measurements/week per patient, patient assessment of ease of use, difficulties encountered, and usefulness.
- The trend of secondary outcomes (QOL, psychological state, and caregiver care burden) over time (day 1, day 28 and day 56) will be analyzed using a general linear model for repeated measures.
- Comparison between the ButterfLife and Control groups: after analyzing the normality of the variables (Shapiro–Wilk test), the differences between the two groups for secondary outcomes (QOL, psychological state, caregiver care burden, caregiver satisfaction with the care received, number of scheduled visits, unscheduled visits, on-call visits, phone calls received by the doctor and nurse, number of visits to the emergency room, number of hospital admissions and hospitalization) will be analyzed with parametric (Student’s *t*-test) or non-parametric tests (Mann–Whitney test).

## 3. Expected Impact

Although it is premature to allude to the outcomes, this section describes the expected impact of the study, aware that it must wait for the conclusion of the study to obtain the final result. The recent improvements in oncological therapies are progressively increasing the life expectancy of cancer patients [36]. This population constitutes a growing cohort at heightened cardiovascular risk for several reasons: the presence of concurrent risk factors, the pathophysiology of the underlying cancer, and several adverse cardiovascular effects of chemoradiation and other targeted therapies. Optimizing the cardiovascular health of cancer patients is an increasing healthcare priority requiring special considerations. Recent research has focused on the development of cardiovascular risk assessment tools tailored specifically for cancer patients. These tools could be used to identify individuals at high risk, prioritize preventive interventions, and monitor and manage cardiovascular risk in cancer patients over time. Overall, it is clear that the cardiovascular health of cancer patients should be a priority for healthcare providers. The use of telemonitoring assessment tools can help to manage the risk of cardiovascular events in advanced cancer patients with multiple comorbidities.

We expect that the results of this study may improve scientific knowledge regarding the impact of telemonitoring and telemedicine systems on QOL and psychological well-being in advanced cancer patients with relevant cardiovascular and respiratory comorbidities assisted at home.

Several studies regarding telemedicine in advanced cancer patients have regularly assessed psychosocial constructs and QOL [7]. Thus, previous studies and reviews reported a significantly higher QOL in telemedicine and telemonitoring interventions rather than in standard care [37,38]. Patients may receive significant support and comfort if their physicians provide them with timely and ongoing feedback on their health. One reason for the improvement of QOL is the capacity of telemedicine to reduce the geographical barrier between patients and clinical staff [38]. Moreover, the constant and continuous monitoring of vital parameters at every stage of the disease or whenever required could reduce suffering and anxiety, improving QOL. However, in this field, it is difficult to have a clear vision [7], whereas many tools could be included within telehealth and information technology (e.g., mobile phone devices, internet-based interventions, interactive health communication applications, virtual programs of support, and symptom monitoring tools) [8]. Specifically, our feasibility study may help to figure out if this telemonitoring tool is useful for advanced cancer patients assisted at home in order to increase the knowledge about the more appropriate telehealth intervention for this specific group of patients. A previous study suggested that the attention provided by telemedicine to patients with advanced cancer is helpful for maintaining quality home care [39]. In addition to reducing the impact of distance, we expect our practice could foster continuous healthcare delivery and closer communication among physicians, patients and families.

As the disease progresses, clinical condition changes and rapidly worsens; thus, adjustments and orientations are necessary to provide good care [39,40]. The daily and constant monitoring of patients’ vital parameters, albeit remotely, may enable the physician to have a clear and updated overview of the clinical trajectory of the disease and its symptoms. On the other hand, the patient may have the feeling of being constantly supported and assisted, significantly lowering distress and anxiety factors and increasing QOL. Indeed, using telemedicine, patients with significant symptoms of distress may receive a high-quality assessment and management of suffering from the comfort of their home without being compelled to move [41].

We expect a significant level of engagement in terms of the number of self-measurements/week per patient, ease of use, difficulties experienced, and usefulness. In this feasibility study, engagement will be evaluated by some indicators as defined by Perski et al., 2017 (and echoed by Goodman et al., 2022), including the frequency and duration of measurements, depth of device use, and others [8,42].

Furthermore, this telemonitoring intervention may have a positive impact on the caregivers. Improving advanced cancer patients’ QOL and psychological symptomatology may relieve the care burden perceived by caregivers and enhance the family’s satisfaction with the care received from the healthcare team. Indeed, previous studies stated that the caregiver’s QOL is related to the patient’s [43], making clear that a possible strategy for enhancing the caregiver’s QOL is to improve the patient’s [44]. In addition, telemedicine may help family caregivers to maintain their personal habits and professional position and to limit financial consequences due to missed workdays required to accompany their loved ones to medical clinics or hospitals [45].

In the present setting, one of the motivations sustaining the advantages of telehealth implementation is the potential to lower home care costs, as well as to improve and expand access to healthcare services [45]. Telehealth could impact costs by relieving the healthcare team from some clinical activities, such as the monitoring of vital parameters at home and reducing travel and workload [45].

The evaluation of secondary outcomes, such as the intensity of doctor/nurse home visits, phone calls, hospital admissions, and hospitalization days, will return an indirect measure of the impact of this telemonitoring intervention in terms of costs and suitability of resources provided by home care services and the National Health System.

## Figures and Tables

**Table 1:** Inclusion and exclusion criteria for eligible participants.

Inclusion Criteria
Men and women with solid and hematologic malignancies in an advanced stage, i.e., a locally advanced or metastatic cancer disease that is unlikely to be cured or controlled with treatment.
Aged ≥ 18 years.
Able to understand the objectives of the study and sign the informed consent.
Able to speak and understand the Italian language.
Karnofsky Performance Status (KPS) ≥ 50.
Presence of at least one of the following comorbidities: ⋅ Chronic obstructive pulmonary disease (COPD); ⋅ Chronic ischaemic heart disease (CIC) after acute myocardial infarction (AMI); ⋅ Pulmonary emphysema; ⋅ Valvulopathy; ⋅ Systemic arterial hypertension; ⋅ Severe coronary artery disease; ⋅ Respiratory failure related to primary or metastatic lung cancer disease; ⋅ Cardiomyopathy developed after oncological treatment. This symptomatology may be directly related to the oncological disease or independent of it (pre-existing pathology at the time of cancer diagnosis, symptoms occurring as a result of oncological therapies, or age-related onset). The severity of these comorbidities must be relevant to the patient’s clinical profile as expressed in at least one of the following conditions: ⋅ Resting oxygen saturation level <90% in room air or oxygen therapy; ⋅ Systolic/diastolic blood pressure greater than 140/90 mmHg or less than 90/60 mmHg, or on antihypertensive medication; ⋅ Resting heart rate greater than 100 or less than 60 beats per minute or being treated with anti-arrhythmic drugs or the presence of a pacemaker.
**Exclusion criteria**
Patients with a diagnosis of dementia or cognitive impairment hampering the comprehension of the study information and/or signing of informed consent.
End-of-life patients.
Patients refusing to participate.

**Table 2:** Questionnaires during the eight weeks of monitoring time. Supplementary Document 2 includes all the questionnaires administrated to patients and caregivers by the physician on Day 1, Day 28 and Day 56.

Timeline	Day 1	Day 28	Day 56
Patient	EQ-5D-3L DASS-21	EQ-5D-3L DASS-21	EQ-5D-3L DASS-21 Evaluation of the ButterfLife device *
Caregiver	CBI	CBI	CBI FAMCARE-2

EQ-5D-3L: EuroQoL-5D-3L questionnaire; DASS-21: Depression Anxiety Stress Scales-21; CBI: Caregiver Burden Inventory; FAMCARE-2: Family Satisfaction with Advanced Cancer Care-2; * Only for patients in ButterfLife group.

## Data Availability

Data are unavailable at the moment since the recruitment is in progress.

## Supplementary Materials

The following supporting information can be downloaded at: https://www.mdpi.com/article/10.3390/jcm12051922/s1, Supplementary Document 1: Example of ButterfLife physiological parameters report; Supplementary Document 2: Questionnaires for patient, caregiver (Day 1, Day 28, and Day 56), and physician.

## References

[1] Costantini M., Sleeman K.E., Peruselli C., Higginson I.J. (2020). Response and role of palliative care during the COVID-19 pandemic: A national telephone survey of hospices in Italy. Palliat. Med..

[2] Mehta A.K., Smith T.J. (2020). Palliative Care for Patients With Cancer in the COVID-19 Era. JAMA Oncol..

[3] Ting R., Edmonds P., Higginson I.J., Sleeman K.E. (2020). Palliative care for patients with severe COVID-19. BMJ.

[4] Shirke M.M., Shaikh S.A., Harky A. (2020). Implications of Telemedicine in Oncology during the COVID-19 Pandemic. Acta Biomed..

[5] Bitar H., Alismail S. (2021). The role of eHealth, telehealth, and telemedicine for chronic disease patients during COVID-19 pandemic: A rapid systematic review. Digit. Health.

[6] Hanlon P., Daines L., Campbell C., McKinstry B., Weller D., Pinnock H. (2017). Telehealth Interventions to Support Self-Management of Long-Term Conditions: A Systematic Metareview of Diabetes, Heart Failure, Asthma, Chronic Obstructive Pulmonary Disease, and Cancer. J. Med. Internet Res..

[7] Kane K., Kennedy F., Absolom K.L., Harley C., Velikova G. (2020). Quality of life support in advanced cancer—Web and technological interventions: Systematic review and narrative synthesis. BMJ Support. Palliat. Care.

[8] Goodman W., Bagnall A.-M., Ashley L., Azizoddin D., Muehlensiepen F., Blum D., Bennett M.I., Allsop M. (2022). The Extent of Engagement With Telehealth Approaches by Patients With Advanced Cancer: Systematic Review. JMIR Cancer.

[9] Finucane A.M., O’Donnell H., Lugton J., Gibson-Watt T., Swenson C., Pagliari C. (2021). Digital health interventions in palliative care: A systematic meta-review. NPJ Digit. Med..

[10] Sofia M., Pisciotta M., Gervasi F., Oldani S., Marzola A., Angelini C. (2021). Fattibilità ed efficacia della telemedicina nel contesto delle cure palliative domiciliari. Riv It Cure Palliat..

[11] Hancock S., Preston N., Jones H., Gadoud A. (2019). Telehealth in palliative care is being described but not evaluated: A systematic review. BMC Palliat. Care.

[12] Zheng Y., Head B.A., Schapmire T.J. (2016). A Systematic Review of Telehealth in Palliative Care: Caregiver Outcomes. Telemed. e-Health.

[13] Head B.A., Schapmire T.J., Zheng Y. (2017). Telehealth in Palliative Care. J. Hosp. Palliat. Nurs..

[14] Mattioli A.V., Cossarizza A., Boriani G. (2020). COVID-19 pandemic: Usefulness of telemedicine in management of arrhythmias in elderly people. J. Geriatr. Cardiol..

[15] Abelsson T., Morténius H., Bergman S., Karlsson A.-K. (2020). Quality and availability of information in primary healthcare: The patient perspective. Scand. J. Prim. Health Care.

[16] Hall J. (2021). The impact of COVID-19 on critical cardiac care and what is to come postpandemic. Future Cardiol..

[17] Wang Y., Wang Y., Han X., Sun J., Li C., Adhikari B.K., Zhang J., Miao X., Chen Z. (2022). Cardio-Oncology: A Myriad of Relationships Between Cardiovascular Disease and Cancer. Front. Cardiovasc. Med..

[18] Muhandiramge J., Zalcberg J.R., van Londen G.J., Warner E.T., Carr P.R., Haydon A., Orchard S.G. (2022). Cardiovascular Disease in Adult Cancer Survivors: A Review of Current Evidence, Strategies for Prevention and Management, and Future Directions for Cardio-oncology. Curr. Oncol. Rep..

[19] Lyon A.R., López-Fernández T., Couch L.S., Asteggiano R., Aznar M.C., Bergler-Klein J., Boriani G., Cardinale D., Cordoba R., Cosyns B. (2022). 2022 ESC Guidelines on cardio-oncology developed in collaboration with the European Hematology Association (EHA), the European Society for Therapeutic Radiology and Oncology (ESTRO) and the International Cardio-Oncology Society (IC-OS). Eur. Heart J..

[20] Ministero della Salute (2022). Approvazione delle Linee Guida per i Servizi di Telemedicina-Requisiti Funzionali e Livelli di Servizio.

[21] Casadio M., Biasco G., Abernethy A., Bonazzi V., Pannuti R., Pannuti F. (2010). The National Tumor Association Foundation (ANT): A 30 year old model of home palliative care. BMC Palliat. Care.

[22] Ostan R., Varani S., Pannuti F., Pannuti R., Biasco G., Bruera E. End of life care for patients with cancer: Clinical, geographical, and socio-cultural differences. Palliat. Support. Care.

[23] Moscato S., Palumbo P., Sichi V., Giannelli A., Varani S., Chiari L. Objective assessment of pain in uncontrolled environment through electrodermal activity in oncological population. Proceedings of the IASP Virtual Series on Pain and Expo.

[24] Moscato S., Sichi V., Giannelli A., Palumbo P., Ostan R., Varani S., Pannuti R., Chiari L. (2021). Virtual Reality in Home Palliative Care: Brief Report on the Effect on Cancer-Related Symptomatology. Front. Psychol..

[25] Franchini L., Varani S., Ostan R., Bocchi I., Pannuti R., Biasco G., Bruera E. (2021). Home palliative care professionals perception of challenges during the COVID-19 outbreak: A qualitative study. Palliat. Med..

[26] Hajesmaeel-Gohari S., Bahaadinbeigy K. (2021). The most used questionnaires for evaluating telemedicine services. BMC Med. Inform. Decis. Mak..

[27] EuroQol Research Foundation EQ-5D-3L User Guide (Version 6.0). https://euroqol.org/publications/user-guides/.

[28] Scalone L., Cortesi P.A., Ciampichini R., Belisari A., D’Angiolella L.S., Cesana G., Mantovani L.G. (2013). Italian Population-Based Values of EQ-5D Health States. Value Health.

[29] Osman A., Wong J.L., Bagge C.L., Freedenthal S., Gutierrez P.M., Lozano G. (2012). The Depression Anxiety Stress Scales-21 (DASS-21): Further Examination of Dimensions, Scale Reliability, and Correlates. J. Clin. Psychol..

[30] Bottesi G., Ghisi M., Altoè G., Conforti E., Melli G., Sica C. (2015). The Italian version of the Depression Anxiety Stress Scales-21: Factor structure and psychometric properties on community and clinical samples. Compr. Psychiatry.

[31] Novak M., Guest C. (1989). Application of a Multidimensional Caregiver Burden Inventory. Gerontologist.

[32] Aoun S., Bird S., Kristjanson L.J., Currow D. (2010). Reliability testing of the FAMCARE-2 scale: Measuring family carer satisfaction with palliative care. Palliat. Med..

[33] D’Angelo D., Punziano A.C., Mastroianni C., Marzi A., Latina R., Ghezzi V., Piredda M., De Marinis M.G. (2017). Translation and Testing of the Italian Version of FAMCARE-2: Measuring Family Caregivers’ Satisfaction With Palliative Care. J. Fam. Nurs..

[34] Ministero del Lavoro della Salute e delle Politiche Sociali (2007). Classificazione delle Malattie, dei Traumatismi, degli Interventi Chirurgici e delle Procedure Diagnostiche e Terapeutiche-“International Classification of Diseases-9th Revision-Clinical Modification” (Italian version).

[35] Bruera E., Kuehn N., Miller M.J., Selmser P., Macmillan K. (1991). The Edmonton Symptom Assessment System (ESAS): A simple method for the assessment of palliative care patients. J. Palliat. Care.

[36] Raisi-Estabragh Z., Kobo O., Freeman P., Petersen S.E., Kolman L., Miller R.J.H., Roguin A., Van Spall H.G.C., Vuong J., Yang E.H. (2022). Temporal trends in disease-specific causes of cardiovascular mortality amongst patients with cancer in the USA between 1999 and 2019. Eur. Heart J.-Qual. Care Clin. Outcomes.

[37] Chen Y.J., Narsavage G.L., Frick K.D., Petitte T.M. (2016). Home-Telemonitoring Lung Cancer Intervention in Appalachia: A Pilot Study. Int. J. Chronic Dis. Ther..

[38] Pang L., Liu Z., Lin S., Liu Z., Liu H., Mai Z., Liu Z., Chen C., Zhao Q. (2020). The effects of telemedicine on the quality of life of patients with lung cancer: A systematic review and meta-analysis. Ther. Adv. Chronic Dis..

[39] Hennemann-Krause L., Lopes A.J., Araújo J.A., Petersen E.M., Nunes R.A. (2015). The assessment of telemedicine to support outpatient palliative care in advanced cancer. Palliat. Support. Care.

[40] Donnem T., Ervik B., Magnussen K., Andersen S., Pastow D., Andreassen S., Nørstad T., Helbekkmo N., Bremnes R.M., Nordoy T. (2012). Bridging the distance: A prospective tele-oncology study in Northern Norway. Support. Care Cancer.

[41] Tang M., Reddy A. (2022). Telemedicine and Its Past, Present, and Future Roles in Providing Palliative Care to Advanced Cancer Patients. Cancers.

[42] Perski O., Blandford A., West R., Michie S. (2017). Conceptualising engagement with digital behaviour change interventions: A systematic review using principles from critical interpretive synthesis. Transl. Behav. Med..

[43] Gill P., Kaur J.S., Rummans T., Novotny P.J., Sloan J.A. (2003). The hospice patient’s primary caregiver. J. Psychosom. Res..

[44] Clark M.M., Rummans T.A., Sloan J.A., Jensen A., Atherton P.J., Frost M.H., Richardson J.W., Bostwick J.M., Johnson M.E., Hanson J.M. (2006). Quality of Life of Caregivers of Patients With Advanced-Stage Cancer. Am. J. Hosp. Palliat. Med..

[45] Watanabe S.M., Fairchild A., Pituskin E., Borgersen P., Hanson J., Fassbender K. (2013). Improving access to specialist multidisciplinary palliative care consultation for rural cancer patients by videoconferencing: Report of a pilot project. Support. Care Cancer.

