# Supplementary material for: Distance Monitoring of Advanced Cancer Patients with Impaired Cardiac and Respiratory Function Assisted at Home: A Study Protocol in Italy
Source: J Clin Med. 2023 Feb 28;12(5):1922. doi: 10.3390/jcm12051922 (PMC10004121; doi:10.3390/jcm12051922)
Supplement: Supplementary file 1 [file jcm-12-01922-s001.zip › Ostan et al_supplementary materials/Supplementary Document 1.pdf]

IppocraTech

Report 02-01-2023 12:44

The values measured by ButterfLife are processed by VST FIVE software, a certified IIa medical device with certified number 0068/QPZ-DM/184-2020 issued by notified organization number 0068

FONDAZIONE ANT 1978 ONLUS Assistenza Nazionale Tumori

User ID: F A

Age	Gender	Hypertensive	Weight (Kg)	Height (cm)	BMI
44	F	NO	60	169	21.01

Average values measured during the test

Systolic BP	***Diastolic BP***	***Heart Rate***	***Oxygenation***	***Breath Frequency***	***Body T.***	***Ambient T.***
[mmHg]	[mmHg]	[bpm]	[%]	[resp/min]	[°C]	[°C]
(100-140)	*(60-90)*	*(60-100)*	*(95-100)*	*(10-20)*	-	-
**126**	**71**	**76**	**99**	**9 ***	**36.9**	**21.3**

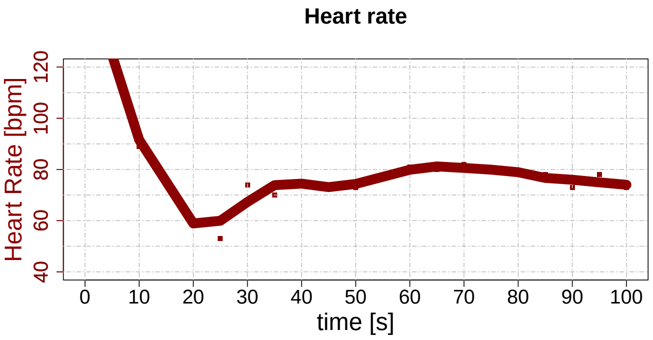

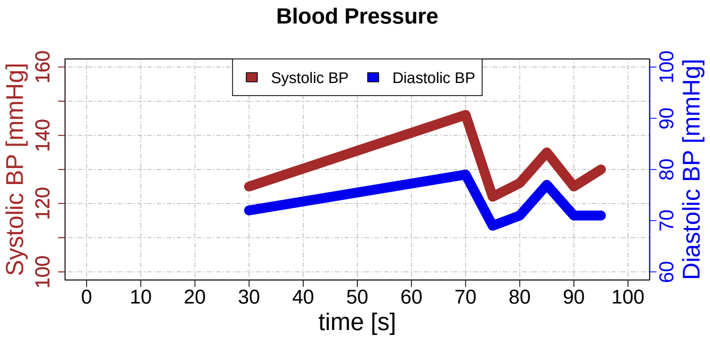

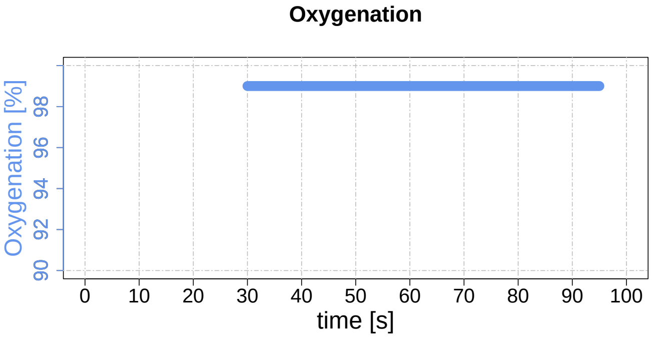

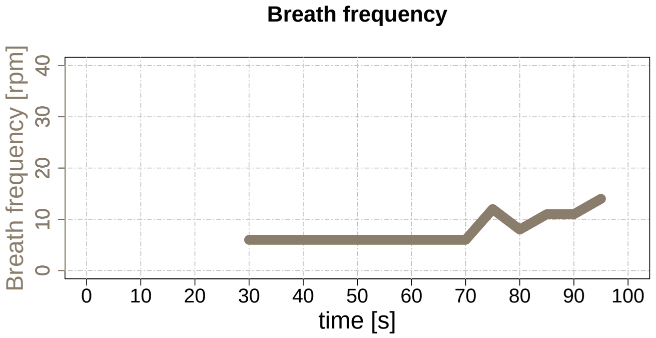

Report created by ButterfLife: 10D07A1DEEF4

IppocraTech® by VST srl – P. IVA: 03752750368 – Via della Scienza 50, 41122 Modena
email: info@ippocra.tech - site: https://ippocra.tech - login: https://my.vital.st
