# Supplementary material for: Distance Monitoring of Advanced Cancer Patients with Impaired Cardiac and Respiratory Function Assisted at Home: A Study Protocol in Italy
Source: J Clin Med. 2023 Feb 28;12(5):1922. doi: 10.3390/jcm12051922 (PMC10004121; doi:10.3390/jcm12051922)
Supplement: Supplementary file 1 [file jcm-12-01922-s001.zip › Ostan et al_supplementary materials/Supplementary Document 2.pdf]

Patient code___________________________ **Date** _________________

Patient data

1. Gender □ Male □ Female

2. Age _______

3. Marital status
- □ Married or Living with a partner
- □ Widower/Widow
- □ Separated/divorced
- □ Single

4. Educational Qualification
- □ No title
- □ Primary school
- □ Secondary school
- □ High School
- □ University degree/diploma or higher qualification

5. Employment
- □ Student
- □ Not employed
- □ Employed
- □ Retired or Off work

6. Who lives in the house with you? (one answer in each line)

	**Yes**	**No**
Nobody, I live alone	□	□
Partner	□	□
Children/grandchildren up to 14 years old	□	□
Children/other relatives aged 15-64	□	□
Other relatives aged 65 and over	□	□
Carer	□	□

7. Do you regularly use the following multimedia technologies?

	**Yes**	**No**
Computer	□	□
Smartphone or tablet	□	□
Video games	□	□

8. Comorbidities

	**Yes**	**No**
Chronic Obstructive Pulmonary Disease	□	□
Chronic ischaemic heart disease after acute myocardial infarction	□	□
Pulmonary emphysema	□	□
Valvulopathy	□	□
Systemic arterial hypertension	□	□
Severe coronary artery disease	□	□
Respiratory failure related to oncologic or metastatic lung disease	□	□
Cardiomyopathy developed after oncological treatment	□	□

Health Questionnaire (EQ-5D-3L)

© 1990 EuroQol Research Foundation. EQ-5D™ is a trade mark of the EuroQol Research Foundation. UK (English) v2.1

Under each heading, please tick the ONE box that best describes your health TODAY.

**MOBILITY**	
I have no problems in walking about	□
I have some problems in walking about	□
I am confined to bed	□
**SELF-CARE**	
I have no problems with self-care	□
I have some problems washing or dressing myself	□
I am unable to wash or dress myself	□
**USUAL ACTIVITIES** (e.g. work, study, housework, family or leisure activities)	
I have no problems with performing my usual activities.	□
I have some problems with performing my usual activities	□
I am unable to perform my usual activities	□
**PAIN/ DISCOMFORT**	
I have no pain or discomfort	□
I have moderate pain or discomfort	□
I have extreme pain or discomfort	□
**ANXIETY/ DEPRESSION**	
I am not anxious or depressed	□
I am moderately anxious or depressed.	□
I am extremely anxious or depressed	□

The best health
you can imagine

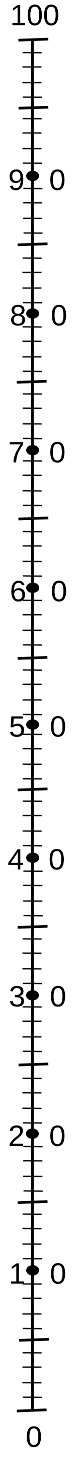

The worst health
you can imagine

We would like to know how good or bad your health is TODAY.

- This scale is numbered from 0 to 100.
- 100 means the best health you can imagine.
  0 means the worst health you can imagine.
- Please mark an X on the scale to indicate how your health is TODAY.
- Now, write the number you marked on the scale in the box below.

YOUR HEALTH TODAY = ☐

DASS – 21

Lovibond, S.H. & Lovibond, P.F. (1995). Manual for the Depression Anxiety & Stress Scales.
(2nd Ed.)Sydney: Psychology Foundation

Please read each statement and circle a number 0, 1, 2 or 3 which indicates how much the statement applied to you over the past week. There are no right or wrong answers. Do not spend too much time on any statement.

The rating scale is as follows:

0	1	2	3
Did not apply to me at all	Applied to me to some degree, or some of the time	Applied to me to a considerable degree or a good part of time	Applied to me very much or most of the time

1	I found it hard to wind down	0	1	2	3
2	I was aware of dryness of my mouth	0	1	2	3
3	I couldn't seem to experience any positive feeling at all	0	1	2	3
4	I experienced breathing difficulty (e.g. excessively rapid breathing, breathlessness in the absence of physical exertion)	0	1	2	3
5	I found it difficult to work up the initiative to do things	0	1	2	3
6	I tended to over-react to situations	0	1	2	3
7	I experienced trembling (e.g. in the hands)	0	1	2	3
8	I felt that I was using a lot of nervous energy	0	1	2	3
9	I was worried about situations in which I might panic and make a fool of myself	0	1	2	3
10	I felt that I had nothing to look forward to	0	1	2	3
11	I found myself getting agitated	0	1	2	3
12	I found it difficult to relax	0	1	2	3
13	I felt down-hearted and blue	0	1	2	3
14	I was intolerant of anything that kept me from getting on with what I was doing	0	1	2	3
15	I felt I was close to panic	0	1	2	3
16	I was unable to become enthusiastic about anything	0	1	2	3
17	I felt I wasn't worth much as a person	0	1	2	3
18	I felt that I was rather touchy	0	1	2	3
19	I was aware of the action of my heart in the absence of physical exertion (e.g. sense of heart rate increase, heart missing a beat)	0	1	2	3
20	I felt scared without any good reason	0	1	2	3
21	I felt that life was meaningless	0	1	2	3

Caregiver data

9. Gender □ Male □ Female

10. Age ________

11. Marital status

□ Married or Living with a partner
□ Widower/Widow
□ Separated/Divorced
□ Single

12. Educational qualification

□ No title
□ Primary school
□ Secondary school
□ High School
□ University degree/diploma or higher qualification

13. Employment

□ Student
□ Not employed
□ Employed
□ Retired or Off work

14. What is your relationship with the patient?

□ Partner
□ Daughter/Son
□ Parent
□ Other, specify ___________________________

CAREGIVER BURDEN INVENTORY (CBI)

Novak, M., & Guest, C. (1989). Application of a multidimensional caregiver burden inventory. *The gerontologist*, *29*(6), 798-803

The questions refer to you providing care for your relative who is suffering from illness;
Choose the number that best represents how often the statement describes your feelings.

0 = not at all   1 = a little   2 = moderately   3 = much   4 = very much

		Items	Score				
T	1	My care receiver needs my help to perform many daily tasks.	0	1	2	3	4
T	2	My care receiver is dependent on me	0	1	2	3	4
T	3	I have to watch my care receiver constantly	0	1	2	3	4
T	4	I have to help my care receiver with many basic functions	0	1	2	3	4
T	5	I don't have a minute's break from my caregiving chores	0	1	2	3	4
S	6	I feel that I'm missing out on life.	0	1	2	3	4
S	7	I wish I could escape from this situation	0	1	2	3	4
S	8	My social life has suffered	0	1	2	3	4
S	9	I feel emotionally drained, due to caring for my care receiver.	0	1	2	3	4
S	10	I expected that things would be different at this point in my life	0	1	2	3	4
F	11	I'm not getting enough sleep	0	1	2	3	4
F	12	My health has suffered	0	1	2	3	4
F	13	Caregiving has made me physically ill	0	1	2	3	4
F	14	I'm physically tired	0	1	2	3	4
D	15	I don't get along with other family members as well as I used to	0	1	2	3	4
D	16	My caregiving efforts aren't appreciated by others in my family	0	1	2	3	4
D	17	I've had problems with my marriage	0	1	2	3	4
D	18	I don't do as good a job at work as I used to	0	1	2	3	4
D	19	I feel resentful of other relatives who could but do not help	0	1	2	3	4
E	20	I feel embarrassed by my care receiver's behavior	0	1	2	3	4
E	21	I feel ashamed of my care receiver	0	1	2	3	4
E	22	I resent my care receiver	0	1	2	3	4
E	23	I feel uncomfortable when I have friends over	0	1	2	3	4
E	24	I feel angry about my reactions toward my care receive	0	1	2	3	4

Total Score ____________

Patient code___________________________ **Date** _________________

Health Questionnaire (EQ-5D-3L)

© 1990 EuroQol Research Foundation. EQ-5D™ is a trade mark of the EuroQol Research Foundation. UK (English) v2.1

Under each heading, please tick the ONE box that best describes your health TODAY.

**MOBILITY**	
I have no problems in walking about	☐
I have some problems in walking about	☐
I am confined to bed	☐
**SELF-CARE**	
I have no problems with self-care	☐
I have some problems washing or dressing myself	☐
I am unable to wash or dress myself	☐
**USUAL ACTIVITIES** (*e.g. work, study, housework, family or leisure activities)*	
I have no problems with performing my usual activities.	☐
I have some problems with performing my usual activities	☐
I am unable to perform my usual activities	☐
**PAIN/ DISCOMFORT**	
I have no pain or discomfort	☐
I have moderate pain or discomfort	☐
I have extreme pain or discomfort.	☐
**ANXIETY/ DEPRESSION**	
I am not anxious or depressed	☐
I am moderately anxious or depressed.	☐
I am extremely anxious or depressed.	☐

The best health
you can imagine

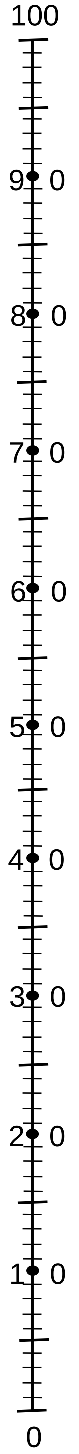

The worst health
you can imagine

We would like to know how good or bad your health is TODAY.

- This scale is numbered from 0 to 100.
- 100 means the best health you can imagine.
  0 means the worst health you can imagine.
- Please mark an X on the scale to indicate how your health is TODAY.
- Now, write the number you marked on the scale in the box below.

YOUR HEALTH TODAY =

DASS – 21

Lovibond, S.H. & Lovibond, P.F. (1995). Manual for the Depression Anxiety & Stress Scales. (2nd Ed.)Sydney: Psychology Foundation

Please read each statement and circle a number 0, 1, 2 or 3 which indicates how much the statement applied to you over the past week. There are no right or wrong answers. Do not spend too much time on any statement.

The rating scale is as follows:

0	1	2	3
Did not apply to me at all	Applied to me to some degree, or some of the time	Applied to me to a considerable degree or a good part of time	Applied to me very much or most of the time

1	I found it hard to wind down	0	1	2	3
2	I was aware of dryness of my mouth	0	1	2	3
3	I couldn't seem to experience any positive feeling at all	0	1	2	3
4	I experienced breathing difficulty (e.g. excessively rapid breathing, breathlessness in the absence of physical exertion)	0	1	2	3
5	I found it difficult to work up the initiative to do things	0	1	2	3
6	I tended to over-react to situations	0	1	2	3
7	I experienced trembling (e.g. in the hands)	0	1	2	3
8	I felt that I was using a lot of nervous energy	0	1	2	3
9	I was worried about situations in which I might panic and make a fool of myself	0	1	2	3
10	I felt that I had nothing to look forward to	0	1	2	3
11	I found myself getting agitated	0	1	2	3
12	I found it difficult to relax	0	1	2	3
13	I felt down-hearted and blue	0	1	2	3
14	I was intolerant of anything that kept me from getting on with what I was doing	0	1	2	3
15	I felt I was close to panic	0	1	2	3
16	I was unable to become enthusiastic about anything	0	1	2	3
17	I felt I wasn't worth much as a person	0	1	2	3
18	I felt that I was rather touchy	0	1	2	3
19	I was aware of the action of my heart in the absence of physical exertion (e.g. sense of heart rate increase, heart missing a beat)	0	1	2	3
20	I felt scared without any good reason	0	1	2	3
21	I felt that life was meaningless	0	1	2	3

CAREGIVER BURDEN INVENTORY (CBI)

Novak, M., & Guest, C. (1989). Application of a multidimensional caregiver burden inventory.
The gerontologist, *29*(6), 798-803

The questions refer to you providing care for your relative who is suffering from illness;
Choose the number that best represents how often the statement describes your feelings.

0 = not at all 1 = a little 2 = moderately 3 = much 4 = very much

		Items	Score				
T	1	My care receiver needs my help to perform many daily tasks.	0	1	2	3	4
T	2	My care receiver is dependent on me	0	1	2	3	4
T	3	I have to watch my care receiver constantly	0	1	2	3	4
T	4	I have to help my care receiver with many basic functions	0	1	2	3	4
T	5	I don't have a minute's break from my caregiving chores	0	1	2	3	4
S	6	I feel that I'm missing out on life.	0	1	2	3	4
S	7	I wish I could escape from this situation	0	1	2	3	4
S	8	My social life has suffered	0	1	2	3	4
S	9	I feel emotionally drained, due to caring for my care receiver.	0	1	2	3	4
S	10	I expected that things would be different at this point in my life	0	1	2	3	4
F	11	I'm not getting enough sleep	0	1	2	3	4
F	12	My health has suffered	0	1	2	3	4
F	13	Caregiving has made me physically ill	0	1	2	3	4
F	14	I'm physically tired	0	1	2	3	4
D	15	I don't get along with other family members as well as I used to	0	1	2	3	4
D	16	My caregiving efforts aren't appreciated by others in my family	0	1	2	3	4
D	17	I've had problems with my marriage	0	1	2	3	4
D	18	I don't do as good a job at work as I used to	0	1	2	3	4
D	19	I feel resentful of other relatives who could but do not help	0	1	2	3	4
E	20	I feel embarrassed by my care receiver's behavior	0	1	2	3	4
E	21	I feel ashamed of my care receiver	0	1	2	3	4
E	22	I resent my care receiver	0	1	2	3	4
E	23	I feel uncomfortable when I have friends over	0	1	2	3	4
E	24	I feel angry about my reactions toward my care receive	0	1	2	3	4

Total Score ____________

Patient code___________________________ **Date** _________________

Health Questionnaire (EQ-5D-3L)

© 1990 EuroQol Research Foundation. EQ-5D™ is a trade mark of the EuroQol Research Foundation. UK (English) v2.1

Under each heading, please tick the ONE box that best describes your health TODAY.

**MOBILITY**	
I have no problems in walking about	☐
I have some problems in walking about	☐
I am confined to bed	☐
**SELF-CARE**	
I have no problems with self-care	☐
I have some problems washing or dressing myself	☐
I am unable to wash or dress myself	☐
**USUAL ACTIVITIES** (*e.g. work, study, housework, family or leisure activities)*	
I have no problems with performing my usual activities.	☐
I have some problems with performing my usual activities	☐
I am unable to perform my usual activities	☐
**PAIN/ DISCOMFORT**	
I have no pain or discomfort	☐
I have moderate pain or discomfort	☐
I have extreme pain or discomfort.	☐
**ANXIETY/ DEPRESSION**	
I am not anxious or depressed	☐
I am moderately anxious or depressed.	☐
I am extremely anxious or depressed.	☐

The best health
you can imagine

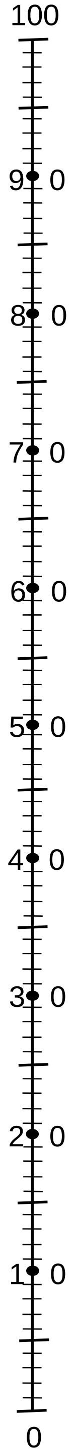

The worst health
you can imagine

We would like to know how good or bad your health is TODAY.

- This scale is numbered from 0 to 100.
- 100 means the best health you can imagine.
  0 means the worst health you can imagine.
- Please mark an X on the scale to indicate how your health is TODAY.
- Now, write the number you marked on the scale in the box below.

YOUR HEALTH TODAY = [ ]

DASS – 21

Lovibond, S.H. & Lovibond, P.F. (1995). Manual for the Depression Anxiety & Stress Scales.
(2nd Ed.)Sydney: Psychology Foundation

Please read each statement and circle a number 0, 1, 2 or 3 which indicates how much the statement applied to you over the past week. There are no right or wrong answers. Do not spend too much time on any statement.

The rating scale is as follows:

0	1	2	3
Did not apply to me at all	Applied to me to some degree, or some of the time	Applied to me to a considerable degree or a good part of time	Applied to me very much or most of the time

1	I found it hard to wind down	0	1	2	3
2	I was aware of dryness of my mouth	0	1	2	3
3	I couldn’t seem to experience any positive feeling at all	0	1	2	3
4	I experienced breathing difficulty (e.g. excessively rapid breathing, breathlessness in the absence of physical exertion)	0	1	2	3
5	I found it difficult to work up the initiative to do things	0	1	2	3
6	I tended to over-react to situations	0	1	2	3
7	I experienced trembling (e.g. in the hands)	0	1	2	3
8	I felt that I was using a lot of nervous energy	0	1	2	3
9	I was worried about situations in which I might panic and make a fool of myself	0	1	2	3
10	I felt that I had nothing to look forward to	0	1	2	3
11	I found myself getting agitated	0	1	2	3
12	I found it difficult to relax	0	1	2	3
13	I felt down-hearted and blue	0	1	2	3
14	I was intolerant of anything that kept me from getting on with what I was doing	0	1	2	3
15	I felt I was close to panic	0	1	2	3
16	I was unable to become enthusiastic about anything	0	1	2	3
17	I felt I wasn’t worth much as a person	0	1	2	3
18	I felt that I was rather touchy	0	1	2	3
19	I was aware of the action of my heart in the absence of physical exertion (e.g. sense of heart rate increase, heart missing a beat)	0	1	2	3
20	I felt scared without any good reason	0	1	2	3
21	I felt that life was meaningless	0	1	2	3

ButterfLife Home device evaluation questionnaire

1. Was the purpose of the study and the use of the device easy to understand?
□ Yes
□ Quite
□ No

2. Was the device easy to use?
□ Yes
□ Quite
□ No

3. Did you have difficulty using the device due to its characteristics (shape, weight, etc.)?
□ Yes
□ Sometimes
□ No

4. Did you have difficulty using the device due to your health condition?
□ Yes
□ Sometimes
□ No

5. Did you find the device was a useful system for monitoring your health condition?
□ Yes
□ Quite
□ No

6. Did you find the device was a useful system for interacting with health care professionals?
□ Yes
□ Quite
□ No

7. Would you like to continue using it in the future to monitor your physiological parameters?
□ Yes
□ No
□ I don’t know

CAREGIVER BURDEN INVENTORY (CBI)

Novak, M., & Guest, C. (1989). Application of a multidimensional caregiver burden inventory. *The gerontologist*, *29*(6), 798-803

The questions refer to you providing care for your relative who is suffering from illness;
Choose the number that best represents how often the statement describes your feelings.

0 = not at all 1 = a little 2 = moderately 3 = much 4 = very much

		Items	Score				
T	1	My care receiver needs my help to perform many daily tasks.	0	1	2	3	4
T	2	My care receiver is dependent on me	0	1	2	3	4
T	3	I have to watch my care receiver constantly	0	1	2	3	4
T	4	I have to help my care receiver with many basic functions	0	1	2	3	4
T	5	I don't have a minute's break from my caregiving chores	0	1	2	3	4
S	6	I feel that I'm missing out on life.	0	1	2	3	4
S	7	I wish I could escape from this situation	0	1	2	3	4
S	8	My social life has suffered	0	1	2	3	4
S	9	I feel emotionally drained, due to caring for my care receiver.	0	1	2	3	4
S	10	I expected that things would be different at this point in my life	0	1	2	3	4
F	11	I'm not getting enough sleep	0	1	2	3	4
F	12	My health has suffered	0	1	2	3	4
F	13	Caregiving has made me physically ill	0	1	2	3	4
F	14	I'm physically tired	0	1	2	3	4
D	15	I don't get along with other family members as well as I used to	0	1	2	3	4
D	16	My caregiving efforts aren't appreciated by others in my family	0	1	2	3	4
D	17	I've had problems with my marriage	0	1	2	3	4
D	18	I don't do as good a job at work as I used to	0	1	2	3	4
D	19	I feel resentful of other relatives who could but do not help	0	1	2	3	4
E	20	I feel embarrassed by my care receiver's behavior	0	1	2	3	4
E	21	I feel ashamed of my care receiver	0	1	2	3	4
E	22	I resent my care receiver	0	1	2	3	4
E	23	I feel uncomfortable when I have friends over	0	1	2	3	4
E	24	I feel angry about my reactions toward my care receive	0	1	2	3	4

Total Score ____________

FAMCARE-2

(Neko, C. FAMCARE and FAMCARE-2 Guidelines.)

Think about the care your family member has receveid on our Pallaitive Care unit. Please answer th e questions below indicating how satisfied you are with the care receveid: very satisfied (VS), satisfied (S), undecided (U), dissatisfied (D), very dissatisfied (VD). Please circle the letters below that best match your experience. We specify that by "Palliative Care Team," a term you will find repeated in the questionnaire, we mean the ANT team that is caring for your Family Member at home.

How satisfied are you with:

The patient's comfort	VS	S	U	D	VD
The way in which the patient's condition and likely progress have been explained by the palliative care team	VS	S	U	D	VD
Information given about the side effects of treatment	VS	S	U	D	VD
The way in which the palliative care team respects the patient's dignity	VS	S	U	D	VD
Meetings with the palliative care team to discuss the patient's condition and plan of care	VS	S	U	D	VD
Speed with which symptoms are treated	VS	S	U	D	VD
Palliative care team's attention to the patient's description of symptoms .	VS	S	U	D	VD
The way in which the patient's physical needs for comfort are met	VS	S	U	D	VD
Availability of the palliative care team to the family	VS	S	U	D	VD
Emotional support provided to family members by the palliative care team	VS	S	U	D	VD
The practical assistance provided by the palliative care team (e.g. bathing, home care, respite)	VS	S	U	D	VD
The Doctor's attention to the patient's symptoms	VS	S	U	D	VD
The way the family is included in treatment and care decisions	VS	S	U	D	VD
Information given about how to manage the patient's symptoms (e.g. pain, constipation)	VS	S	U	D	VD
How effectively the palliative care team manages the patient's symptoms	VS	S	U	D	VD
The palliative care team's response to changes in the patient's care needs	VS	S	U	D	VD
Emotional support provided to the patient by the palliative care team	VS	S	U	D	VD

Date ____/____/________

1. **Gender** ◻ Male ◻ Female

2. **Age** _______

3. **Occupation**

◻ Doctor
◻ Nurse
◻ Psychologist
◻ Other________________

4. **Informatics knowledge**

◻ Poor
◻ Good
◻ Excellent

5. **Did you read the user's guide before you started configuring the device?**

◻ Yes
◻ No

6. **Did you read the user's guide before you started using the device?**

◻ Yes
◻ No

**Interface assessment**	**Answer** 0 equals "I did not understand how to do it" and 4 equals "I succeeded in the operation without any problems"				
Ease of set-up	0	1	2	3	4
Understanding of device features	0	1	2	3	4
Ease of understanding instructions	0	1	2	3	4
Was I able to use the device without any problems?	0	1	2	3	4
Understanding device labeling	0	1	2	3	4
Understanding of errors that may have shown up	0	1	2	3	4

Free notes and ideas for improvement

____
____
____
____
____
